# Mitochondrial Lon-induced mtDNA leakage contributes to PD-L1–mediated immunoescape via STING-IFN signaling and extracellular vesicles

**DOI:** 10.1136/jitc-2020-001372

**Published:** 2020-12-02

**Authors:** An Ning Cheng, Li-Chun Cheng, Cheng-Liang Kuo, Yu Kang Lo, Han-Yu Chou, Chung-Hsing Chen, Yi-Hao Wang, Tsung-Hsien Chuang, Shih-Jung Cheng, Alan Yueh-Luen Lee

**Affiliations:** 1 National Institute of Cancer Research, National Health Research Institutes, Zhunan, Miaoli, Taiwan; 2 Immunology Research Center, National Health Research Institutes, Zhunan, Miaoli, Taiwan; 3 School of Dentistry, National Taiwan University, Taipei, Taiwan; 4 Department of Dentistry, National Taiwan University Hospital, College of Medicine, National Taiwan University, Taipei, Taiwan; 5 Department of Biotechnology, College of Life Science, Kaohsiung Medical University, Kaohsiung, Taiwan; 6 Graduate Institute of Biomedical Sciences, China Medical University, Taichung, Taiwan

**Keywords:** tumor microenvironment, tumor biomarkers, interferon inducers, tumor escape, inflammation

## Abstract

**Background:**

Mitochondrial Lon is a chaperone and DNA-binding protein that functions in protein quality control and stress response pathways. The level of Lon regulates mitochondrial DNA (mtDNA) metabolism and the production of mitochondrial reactive oxygen species (ROS). However, there is little information in detail on how mitochondrial Lon regulates ROS-dependent cancer immunoescape through mtDNA metabolism in the tumor microenvironment (TME).

**Methods:**

We explored the understanding of the intricate interplay between mitochondria and the innate immune response in the inflammatory TME.

**Results:**

We found that oxidized mtDNA is released into the cytosol when Lon is overexpressed and then it induces interferon (IFN) signaling via cGAS-STING-TBK1, which upregulates PD-L1 and IDO-1 expression to inhibit T-cell activation. Unexpectedly, upregulation of Lon also induces the secretion of extracellular vehicles (EVs), which carry mtDNA and PD-L1. Lon-induced EVs further induce the production of IFN and IL-6 from macrophages, which attenuates T-cell immunity in the TME.

**Conclusions:**

The levels of mtDNA and PD-L1 in EVs in patients with oral cancer function as a potential diagnostic biomarker for anti-PD-L1 immunotherapy. Our studies provide an insight into the immunosuppression on mitochondrial stress and suggest a therapeutic synergy between anti-inflammation therapy and immunotherapy in cancer.

## Background

Mitochondria are traditionally considered as a cellular energy generator and apoptosis mediator. More recently, mitochondria have been demonstrated to have important roles in immune responses.^1^ First, mitochondria act as a major source of endogenous reactive oxygen species (ROS) that escape from the metabolic process and the electron transport chain during oxidative phosphorylation.^2 3^ Mild levels of ROS and prolonged ROS production are considered to lead to chronic inflammation. Inflammatory cytokines and signaling pathways, such as NF-κB and TGF-β, are activated by ROS, which is linked to infectious diseases and cancer progression.^4–6^ Second, mitochondria orchestrate signaling and effector functions to activate immune cells and antimicrobial defense.^7^ Microbial signs of infection are detected by cells by pattern recognition receptors to initiate innate immune and inflammatory cascades. The receptors recognize a broad type of biochemical molecules that originate from the pathogen-associated molecular patterns (PAMPs) or from stress-induced damage-associated molecular patterns (DAMPs). Recent data have clearly revealed that mitochondria are major sources of DAMPs, and mitochondrial DNA (mtDNA) is especially crucial for mitochondrial DAMPs that induce host immune responses.^8^ mtDNA fragments from the mitochondria into the cytosol can be detected by cytosolic DNA sensors such as cGAS (cyclic GMP-AMP synthase),^9 10^ which result in innate immune activation. Emerging studies characterized the expanding roles for mtDNA as an endogenous trigger of both pro-inflammatory and cGAS-STING (Stimulator of Interferon Genes)–dependent interferon (IFN) responses.^11^

Mitochondrial Lon plays a crucial role in protein quality control, metabolism, mitophagy, and stress response, which maintains the normal function, biogenesis, and homeostasis of mitochondria.^12 13^ Lon is a multiple-function protein that has ATP-dependent proteolytic,^12^ chaperone,^14–16^ and DNA-binding activity.^17 18^ The studies show that Lon plays an important role in mtDNA maintenance and expression, and the strains lacking mitochondrial Lon suffer large deletions in mtDNA in *Saccharomyces cerevisiae*.^19 20^ Consistently, mitochondrial Lon is associated with the components of mitochondrial nucleoids, mtDNA polymerase γ, Twinkle helicase, and TFAM (mitochondrial transcription factor A),^18^ and the phosphorylated TFAM is degraded by Lon protease.^21^ These findings further support the notion that Lon participates directly in mtDNA metabolism, damage, and repair.

Furthermore, mitochondrial Lon is a stress protein and induced by various stresses such as unfolded protein response, hypoxia, and oxidative stress.^22 23^ We also found that mitochondrial ROS generation induced by Lon is involved in the function of Complex I, NADH dehydrogenase, and PYCR1 enzyme.^5 22^ On oxidative stress, Lon upregulation is required for cancer cell survival, metastasis, and tumorigenesis through p38-NF-κB–dependent pathways.^5 22 24^ These results indicated that mitochondrial Lon acts as a ROS regulator to induce the inflammatory response that further promotes the secretion of TGF-β, IL-13, and IL-6, eventually triggering M2 macrophage polarization in the tumor microenvironment (TME).^5^ Moreover, mitochondrial ROS causes direct damages to metabolic enzymes, lipids in the membrane, and mtDNA, and mtDNA damages and mutations contribute to tumorigenesis.^25 26^ Although mitochondrial Lon showed a reduced binding ability to mtDNA on oxidative stress,^27^ the underlying mechanism of Lon-induced ROS in inflammation and immunoescape through mtDNA integrity has not been fully elucidated yet.

Here, we show that mitochondrial Lon-induced ROS trigger mtDNA damages and oxidized mtDNA is released into the cytosol in cancer. The mtDNA induces IFN signaling via cGAS-STING-TBK1, which upregulates PD-L1 and IDO-1 expression to inhibit T-cell activation. In addition, the upregulation of Lon induces the secretion of extracellular vehicles (EVs), which carry mtDNA and PD-L1. Lon-induced EVs further induce the production of IFN and IL-6 from macrophages, which attenuates innate and CD8^+^ T-cell immunity in the TME. Our studies demonstrate that mitochondrial Lon-induced mtDNA leakage and PD-L1 in EVs are possible mechanisms to create an immunosuppressive TME that promotes tumorigenesis. The levels of mtDNA and PD-L1 in EVs in patients with oral cancer may function as a potential diagnostic biomarker for anti-PD-L1 immunotherapy.

### Patients and clinical sample

Serum specimens of patients with oral squamous cell carcinoma (OSCC) were used for mtDNA and ELISA analysis based on the availability of diagnostic specimens from the Department of Dentistry at National Taiwan University Hospital, Taipei, Taiwan with approval from the Institutional Review Board. All experiments were performed in accordance with relevant guidelines and regulations.

### Cell culture and cell treatment

OEC-M1, HSC-3, DOK, 3B11, RAW264.7, and B16/F10 (mouse melanoma) cells were cultured in medium containing Dulbecco’s modified Eagle’s essential medium (DMEM) (GIBCO, New York, NY, USA), supplemented with 10% fetal bovine serum (FBS qualified; Invitrogen) and penicillin/streptomycin (50 U/mL; Sigma, St. Louis, MO, USA). THP1 were cultured in RPMI1640 medium with 10% FBS.

### Reagents and antibodies

Antibodies to human Lon were produced as described previously.^28^ The antibodies used in this study were purchased as indicated: antibodies to PD-L1, CD63, TSG101, and GAPDH from Gentex (Hsinchu, Taiwan); TFAM (D5C8), TBK1, Phospho-TBK1 (Ser172), Phospho-IRF3 (Ser396), and IDO1 from Cell Signaling Technology (Beverly, MA, USA). Myc tag was obtained from Merck Millipore; HSP70 was obtained from Thermo Fisher Scientific; Nuclease P1, Alkaline phosphatase, and dideoxycytidine (ddC) were obtained from Sigma Aldrich (St. Louis, MO, USA); 8-OHdG antibody (E-8) was purchased from Santa Cruz Biotech (Dallas, TX, USA). CPG 2007 was a gift from Dr. Tsung-Hsien Chuang (Immunology Research Center, National Health Research Institutes, Taiwan). ELISA kits for the detection of human and murine cytokines were purchased from Abcam (USA), and luciferase assay reagents were purchased from Promega (Madison, WI, USA).

### Immunofluorescence staining

Please see online supplemental methods for the detail.

10.1136/jitc-2020-001372.supp1 Supplementary data

### ELISA for genomic 8-OHdG level quantification

HSC3 cells were collected and their genomic DNA was isolated using Tissue & Cell genomic DNA purification kit (GeneMark). Two-microgram DNA samples were converted from dsDNA to ssDNA by heat denaturation, followed by digestion to single nucleotides using nuclease P1 (1 U for 2 hours at 50°C). Finally, single nucleotides are converted to single nucleosides by alkaline phosphatase (1 U for 1 hour at 37°C). Digested genomic DNA was performed by ELISA kit (Abcam, ab201734). The determination range was 0.9375 to 60 ng/mL. The values from each DNA sample were calculated based on calibration sigmoid plots of standard 8-OH-dG at various concentrations by fitting a logistic curve (R^2^ >0.99).

### Measurement of mitochondrial DNA common deletion (CD)

The HSC3 cells were transiently transfected with vector or Lon using MaestroFectin in vitro Transfection reagent (Omics Bio, Taipei City, Taiwan) according to the manufacturer’s protocol, and the cells were collected and their genomic DNA was isolated using Tissue & Cell genomic DNA purification kit (GeneMark). Measurement of common deletion was performed by PCR using the sequence spanning the CD that was amplified using the following primers: mtDNA 8293–8321: 5′-CCCACTGTAAAGCTAACTTAGCATTAACC and mtDNA 13530–13509: 5′-GGTTTCGATGATGAGGTCTTTG. Mitochondrial DNA deletion-specific primer sets flank the breakpoints such that amplicon of the deletion-containing genomes yields an amplification product of 262 bp. Semi-quantitative PCR reactions were performed at 95°C for 1 min followed by 40 cycles at 95°C for 30 s, 53.5°C for 30 s, 72°C for 30 s, and a final elongation for 3 min. The products were separated by electrophoresis on a 1.8% agarose gel stained with ethidium bromide. CD was normalized to the internal standard (IS) of mtDNA was amplified with the following primers: IS-F, 5′-GATTTGGGTACCACCCAAGTATTG and IS-R, 5′-AATATTCATGGTGGCTGGCAGTA.

### Genomic DNA isolation and long-range PCR

HSC3 cells in the FBS-free DMEM medium were treated with 2 mM H_2_O_2_ for 1 hour at 37°C. After washing with PBS, the media were replaced by a complete culture medium and the cells were collected at the indicated time points post-treatment. Genomic DNA was isolated using Tissue & Cell genomic DNA purification kit (GeneMark). Measurement of repair efficiency of mtDNA was performed using PCR that amplifies long mtDNA (16,261 bp) target. The mitochondrial NADH dehydrogenase 1 (mtND1) was quantified by PCR using the following primers: mtND1-3212F: 5′-CACCCAAGAACAGGGTTTGT; mtND1-3319R: 5′-TGGCCATGGGTATGTTGTTAA. The input of the mtDNA template was adjusted based on mtND1 to ensure that an equal amount of mtDNA template in each sample was used for the long-range PCR reaction. The long mtDNA was quantified using the following primers: mtDNA gene, sense: 5′-TGAGGCCAAATATCATTCTGAGGGGC and antisense: 5′-TTTCATCATGCGGAGATGTTGGATGG. PCR reactions were performed at 95°C for 1 min followed by 30 cycles at 98°C for 10 s, 60°C for 40 s, 68°C for 16 min, and a final elongation for 10 min. The products were separated by electrophoresis on a 0.8% agarose gel stained with ethidium bromide.

### Quantitative PCR (qPCR)

The qPCR was performed by a real-time PCR machine (Applied Biosystems 7900) using the SYBR Green master PCR mix (Applied Biosystems). The primer sequences used are as follows: IFN-α: 5′-GACTCCATCTTGGCTGTGA-3′, 5′- TGATTTCTGCTCTGACAACCT-3; beta-actin: 5′-CTCTTCCAGCCTTCCTTCCT-3′, 5′-AGCACTGTGTTGGCGTACAG-3′; IFNG: 5′-GTATTGCTTTGCGTTGGACA-3′, 5′-GAGTGTGGAGACCATCAAGGA-3′; IRF3: 5′-ACCAGCCGTGGACCAAGAG-3′ and 5′-TACCAAGGCCCTGAGGCAC-3′; human PD-L1: 5′-TGTACCGCTGCATGATCAG-3′ and 5′-AGTTCATGTTCAGAGGTGACTG-3′; mouse PD-L1: 5′-GACCAGCTTTTGAAGGGAAATG and 5′-CTGGTTGATTTTGCGGTATGG-3′; D-loop: 5′-TTTAGACGGGCTCACATCACC-3′ and 5′-TGCGTGCTTGATGCTTGTC-3′; ND4: 5′-GCCCAAGAACTATCAAACTCCTGA-3′ and 5′-CGGCAAGTACTATTGACCCAGC-3′; Cytb: 5′-GCCATCCCATACATTGGGAC-3′ and 5′-CGTGCAAGAATAGGAGGTGGA-3′. All amplifications were performed in triplicate.

### Purification of EVs

EVs were isolated by differential centrifugation as previously described.^29^ Briefly, the culture medium was centrifuged at 300×*g* for 10 min to remove cells and at 3000×*g* and 10,000×*g* to remove cell debris. Then, EVs were pelleted by ultracentrifugation at 120,000×*g* for 90 min and afterward washed by PBS.

### Transmission electron microscopy (TEM)

The experiments were performed as previously described by reported protocols. EVs isolated from the cells were fixed in 2% glutaraldehyde in 0.1 M phosphate buffer, and a 2% phosphotungstic acid solution (pH 7.0) was used for negative staining. Negative staining were used for the single-droplet negative staining technique on continuous and holey carbon support films. All TEM procedures were performed by Bio Materials Analysis Technology (Bio MA-Tek, Taiwan).

### Mouse bone marrow–derived macrophages (BMDMs) and splenocyte preparation

BMDMs and splenocytes were isolated as previously described by reported protocols.^30^ Briefly, BMDMs and splenocytes were collected from male and female C57BL/6 mice 6 to 8 weeks old. To generate the BMDMs, mouse bone marrow cells from mouse tibias and femurs were used. After lysing the red blood cells by ACK buffer (150 mM NH_4_Cl, 1 mM KHCO_3_, 0.1 mM Na_2_EDTA), cells were maintained in complete DMEM with 30% L929 conditioned medium for 5 days, followed by DMEM medium with 10% FBS. Mouse splenocytes were cultured in RPMI1640 medium with 10% FBS.

### Animal studies

Male 6-week-old C57BL/6 mice were used for in vivo tumorigenesis assay. B16-F10 (5×10^4^ cells) and B16-F10-Lon (5×10^4^ cells) suspended in 50 µL of medium with 50 µL of Matrigel basement membrane matrix (BD Biosciences) were injected subcutaneously into the dorsal flank. Intraperitoneally injected mice were treated with PD-L1 (200 µg, Clone 10F.9G2; Bio X Cell) antibody and an isotype control antibody (200 µg, Clone MPC-11; Bio X Cell) every 3 days four times and tumor size measured weekly for 17 days. Tumor volumes are estimated from their length (l) and width (w) using the formula: tumor volume=l×w^2^×0.52. Tumors were collected and fixed in 4% paraformaldehyde with PBS and administered for paraffin histologic analysis. Sections of paraffin-embedded tissues (5 µm) were stained with H&E and for immunofluorescence staining, or frozen in liquid nitrogen for protein extraction.

All studies performed with mice were conducted in accordance with the protocols approved by the Institutional Animal Care and Use Committee of the National Health Research Institutes (approval number: NHRI-IACUC-108049-A).

### TLR9 activation assays

TLR9 activation assays were performed following previously reported protocols.^30^ Briefly, HEK 293 cells were seeded on 24-well plates overnight. Afterward, the cells were co-transfected with the TLR9 expression vector, β-galactosidase plasmid, and the NF-κB-driven luciferase reporter plasmid overnight. Then the transfected cells were incubated with EVs for 8 hours. Afterward, cell lysates were collected and luciferase activity was detected. Relative luciferase activities were calculated compared with untreated control. The data were performed as mean±SD (n=3).

### Isolation and culture of mouse T cells

Mouse T cell was puriﬁed from splenocytes using Mouse T Lymphocyte Enrichment Set-DM (BD Biosciences, USA), according to the manufacturer’s instructions. Purified mouse T lymphocyte are stimulated with plate-bound anti-mouse CD3 (clone 145-2 C11, 25 µg/mL) and soluble anti-mouse CD28 (clone 37.51, 2 µg/mL) for 2 days in culture together with msIL-2 (10 ng/mL). Cells were analyzed by flow cytometry (FACSCalibur; BD) for the expression of CD25, CD3, CD4, and CD8.

### Statistical analysis

All data were performed as the mean±SD of three independent experiments. Statistical analyses were performed using Student’s t-test with a significance level of p <0.05.

## Results

### Mitochondrial Lon-induced oxidative stress persuades mtDNA damage

**Figure 1:**
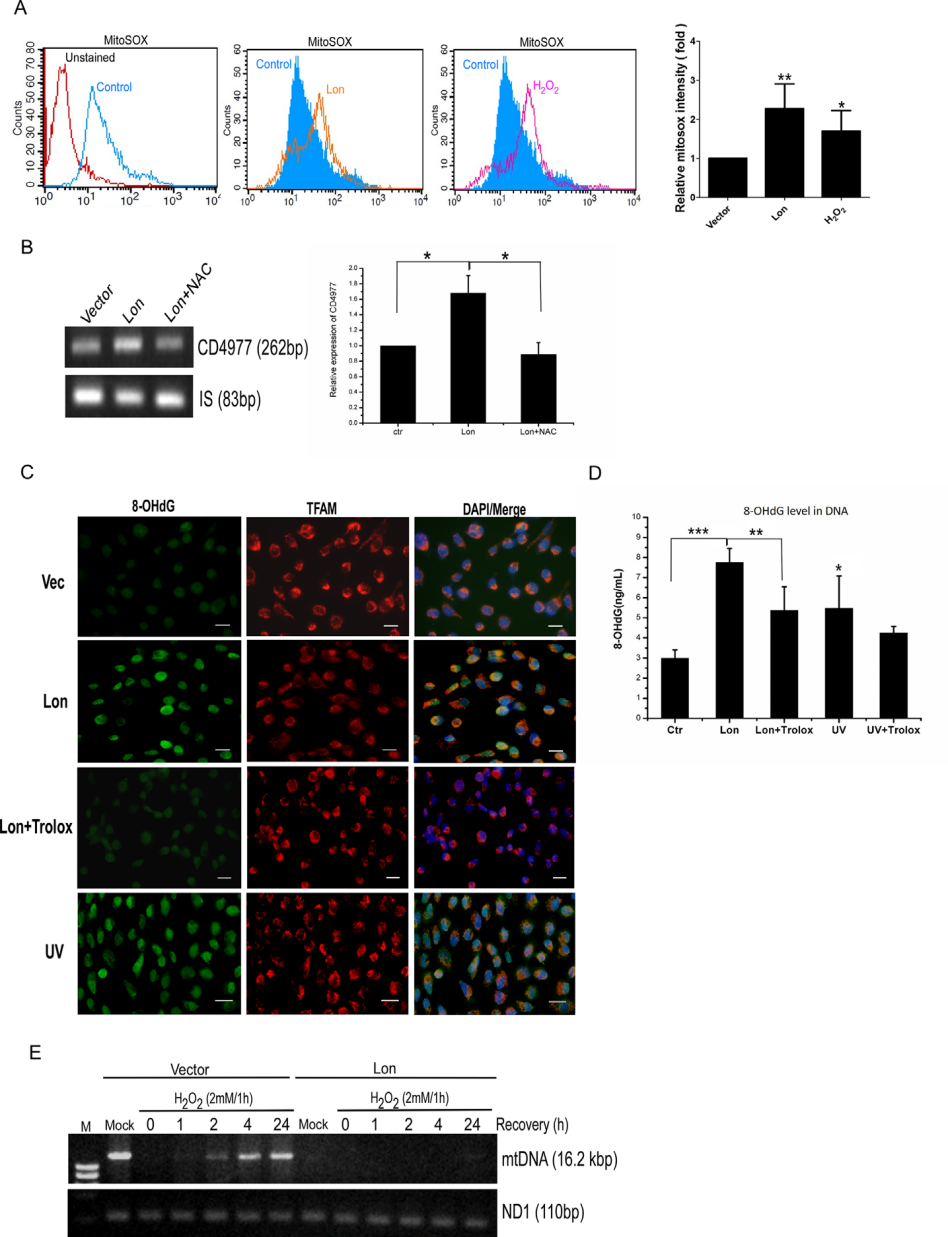
Mitochondrial Lon induces oxidative stress that persuades mitochondrial DNA (mtDNA) damages. (A) Mitochondrial Lon overexpression induces ROS production. Mitochondrial superoxide generation from Lon-overexpressing OECM-1 cells was assessed by flow cytometry after cells were treated with MitoSox. H_2_O_2_ treatment acts as a positive control. The histogram of FACS plots is shown in right panel. The error bars shown in the panel represent the SD from three independent experiments. *p<0.05; **p<0.01. (B) Mitochondrial Lon induces ROS-dependent mtDNA damages of common deletion (CD). The level of common deletion of 4977 bp (CD4977) was semi-quantified by PCR. The HSC3 cells were transiently transfected with vector or Lon for 36 hours and treated with anti-oxidant NAC (2 mM) or not for 1 hour prior to cell collection. Data from three independent experiments. *p<0.05. (C, D) Mitochondrial Lon induces ROS-dependent DNA damages. (C) Mitochondrial Lon-induced oxidative damages of mtDNA and nuclear DNA were verified by immunofluorescence. HSC3 cells were transiently transfected with vector or Lon for 24 hours and treated with Trolox (0.5 mM) for 5 hours or not. The cells were fixed and immunostained by 8-hydroxy-2′-deoxyguanosine (8-OHdG, green) and anti-TFAM (red) antibodies. DNA was stained with DAPI (blue). HSC-3 cells were treated with UV 50 kJ/m^2^ as a positive control. Scale bars=20 µm. (D) Mitochondrial Lon-induced oxidative damages of mtDNA and nuclear DNA were verified by 8-OHdG level through ELISA. HSC3 cells were transiently transfected with vector or Lon for 24 hours and treated with Trolox (0.5 mM) for 5 hours or not. Their genomic DNA was isolated using Tissue & Cell genomic DNA purification kit (GeneMark). The ELISA of 8-OHdG was performed using 2 µg digested genomic DNA samples according to the commercial manufacturer’s protocol. HSC3 cells were treated with UV 50 kJ/m^2^ and allowed 5 hours of recovery. The determination range was 0.9375–60 ng/mL. The error bars shown in the panel represent the SD from three independent experiments. *p<0.05; **p<0.01; ***p<0.001. (E) Mitochondrial Lon overexpression inhibits mtDNA repair capacity. HSC3 cells were transiently transfected with vector or Lon for 24 hours and treated with 2 mM H_2_O_2_ for 1 hour. Then the cells were collected at recovery time points of 0, 1, 2, 4, and 24 hours after H_2_O_2_ treatment. Long-range PCR of full-length mtDNA was performed. An equal amount of mtDNA template in each sample normalized by the mtND1 gene was used for the long-range PCR.

In cancer cells, increased levels of ROS are released from mitochondrial dysfunction, abnormal peroxisome activity, oncogene activity, and metabolic activity. We found previously that increased Lon has been shown to increase mitochondrial ROS production.^22^ To confirm whether ROS were stimulated by overexpressed Lon in OSCC, OEC-M1 cells with Lon overexpression were stained with MitoSOX red to assess mitochondrial ROS level. The result of flow cytometry showed that mitochondrial Lon persuades the production of mitochondrial superoxide anions (figure 1A), confirming that overexpressed Lon increases the production of mitochondrial ROS. To address whether Lon overexpression increases mtDNA damage, CD and oxidative damage of mtDNA were examined. HSC3 cells were transiently transfected with pcDNA3-Lon and then total cellular DNA was isolated. The results of semi-quantitative PCR indicated that Lon-induced ROS increase the mtDNA CD deletions, and *N*-acetyl cysteine (NAC) decreases the CD deletions (figure 1B). To prove that Lon overexpression leads to mtDNA oxidative damage, we checked the accumulation of 8-oxo-2′-dihydroguanine (8-oxo-dG/8-OH-dG) in mtDNA, which is a biomarker of oxidatively damaged DNA.^31^ We performed an 8-OHdG detection experiment using immunofluorescence and ELISA. The results of immunofluorescence and ELISA experiment showed that mitochondrial Lon increases 8-OHdG lesions on both nuclear and mitochondrial DNA, and Trolox, the ROS scavenger, decreases the Lon-induced 8-OHdG level (figure 1C, D). By using TFAM as a mtDNA biomarker, we found that mitochondrial Lon-induced 8-OHdG can be co-localized with TFAM (figure 1C), suggesting that mitochondrial Lon–ROS induces mtDNA oxidative damage. To further assess how increased Lon causes the oxidative damage, we overexpressed Lon to examine the repair efficiency of mtDNA. Long-range PCR was performed to amplify 16.2 kb mtDNA and mtND1 was used to normalize the mtDNA quantity and as a loading control. The results revealed that the overexpressed Lon group shows a higher level of oxidative damage and a lower repair efficiency in mtDNA in response to oxidative stress in OSCC (figure 1E). These results suggest that mitochondrial Lon overexpression promotes oxidative damage of mtDNA via ROS production.

### Mitochondrial Lon overexpression promotes mtDNA release into cytosol under oxidative stress

**Figure 2:**
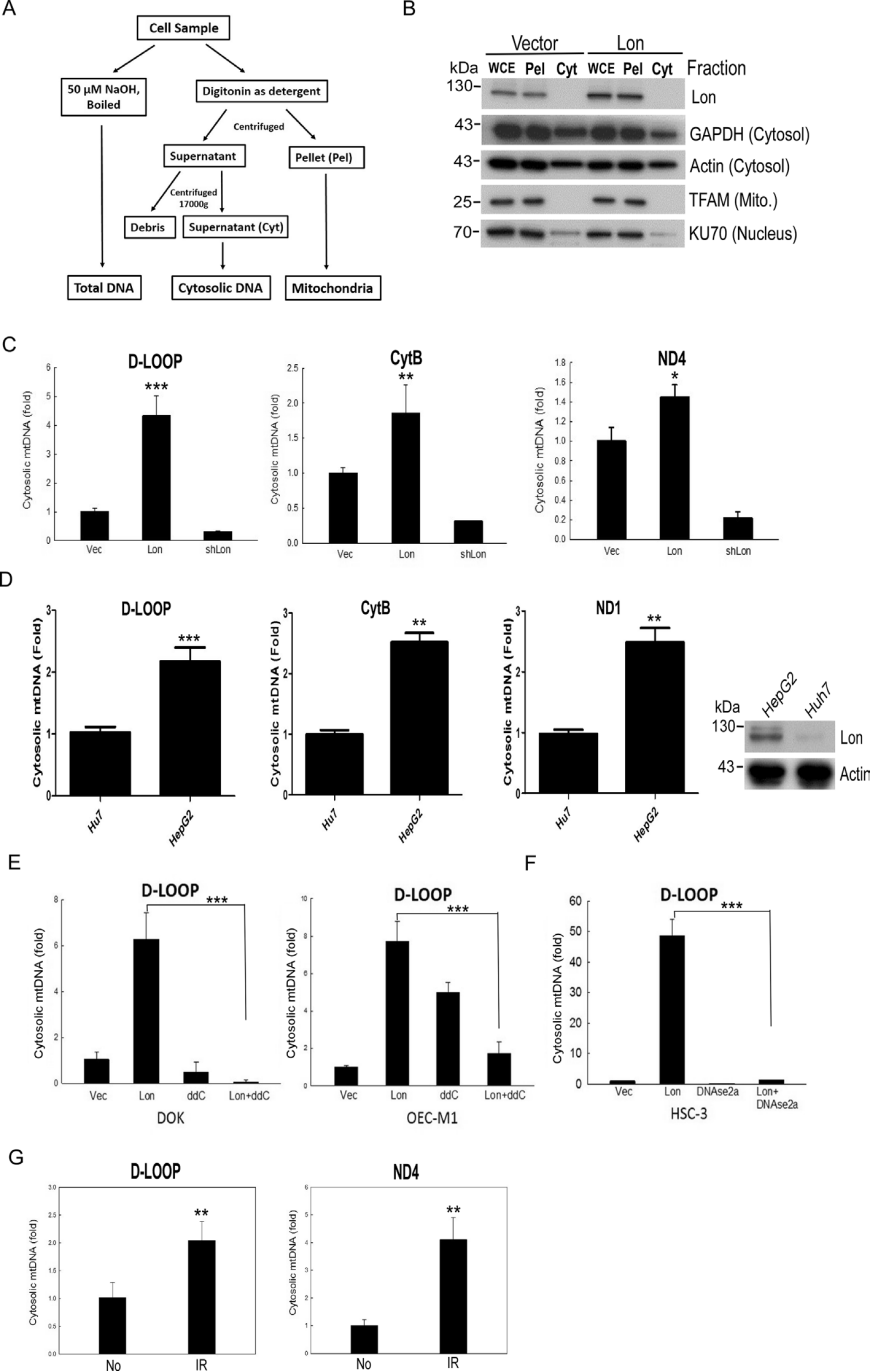
Lon overexpression promotes the accumulation of cytosolic mtDNA. (A) Isolation flowchart of cytosolic mtDNA fraction. (B) Cytosolic and mitochondrial fractions were isolated from oral squamous cell carcinoma (OSCC) cells using the digitonin buffer and confirmed by immunoblotting using the indicated antibodies. Cyt, supernatant; Pel, pellet; WCE, whole cell extract. (C) Mitochondrial Lon induces mtDNA release into the cytosol in OSCC. The ratio of cytoplasmic to total mtDNA was determined by qPCR analysis of DOK cells transfected with the plasmids encoding Lon or Lon-shRNA for 24 hours (n=3). CytB, cytochrome *b*; ND4, NADH dehydrogenase subunit 4. (D) Mitochondrial Lon promotes the mtDNA leakeage to the cytosol in hepatocellular carcinoma. The ratio of cytoplasmic to total mtDNA was determined by qPCR analysis of HepG2 and Huh7 cells. Mitochondrial Lon protein level was confirmed by immunoblotting (right panel). (E, F) The mtDNA leakage induced by Lon is decreased by mtDNA replication inhibitor and DNAse treatment. The ratio of cytoplasmic to total mtDNA was determined by qPCR analysis of OSCC cells transfected with the plasmids encoding vector or Lon and treated with or without 2′,3′-dideoxycytidine (ddC). (E) The ratio of cytoplasmic to total mtDNA was determined by qPCR analysis of OSCC cells transfected with the plasmids encoding vector, Lon, and/or DNAse2a. (F) One-way analysis of variance followed by Student’s t-test. Data are expressed as mean±SEM. ***p<0.001. (G) IR induces the mtDNA leakeage to the cytosol in OEC-M1. The ratio of cytoplasmic to total mtDNA was determined by qPCR analysis of OEC-M1 cells with or without 5 Gy IR exposure (n=3).

To investigate the function of mitochondrial Lon–ROS axis in mtDNA damage that causes tumor progression, we overexpressed Lon in DOK cells and treated them with or without NAC. Using microarray expression analysis, we found that Lon overexpression induces the ROS-dependent inflammatory response in cancer cells,^5^ such as NF-κB and interferon (IFN)–stimulated genes (ISGs)/IFN signalings. Thus, we hypothesized that mitochondrial Lon overexpression triggers IFN signaling via ROS-dependent mtDNA damage and the release of mtDNA into the cytosol in cancer. To test this hypothesis, we first separated and collected the cytosol fraction from the mitochondrial fraction by digitonin buffer (figure 2A), and the fractions were confirmed by Western blotting analysis (figure 2B). Then the cytosol fraction was used to detect the extra mtDNA in the Lon-overexpressing oral cancer cells. Analysis of cytosolic fraction showed a 0.5-fold to 4-fold increase of specific mtDNA fragments from D-loop, ND4, and CytB gene in the Lon-overexpressing oral cancer cells and a decrease in the Lon-knocked-down cells (figure 2C and online supplemental figure S1).

Similar results were observed in hepatoma cells that more mtDNA in the cytosol was detected in HepG2 cells with high Lon expression compared with Huh7 cells (figure 2D). We also observed that the mtDNA in the cytosol was decreased when we treated with mtDNA replication inhibitor (ddC)^32 33^ or overexpressed DNase2a in the Lon-overexpressing OSCC cells (figure 2E, F). Since ionizing irradiation (IR) stimulates ROS production, we further examined whether the irradiation-induced ROS promote the mtDNA release into the cytosol. The results showed that the mtDNA in the cytosol was increased after 5 Gy X-ray irradiation in OSCC cells (figure 2G). The results indicate that the mitochondrial Lon–ROS axis promotes mtDNA release into the cytosol in cancer cells under oxidative stress.

### Lon–mtDNA axis induces IFN signaling through STING-TBK1–dependent pathway on oxidative stress

**Figure 3:**
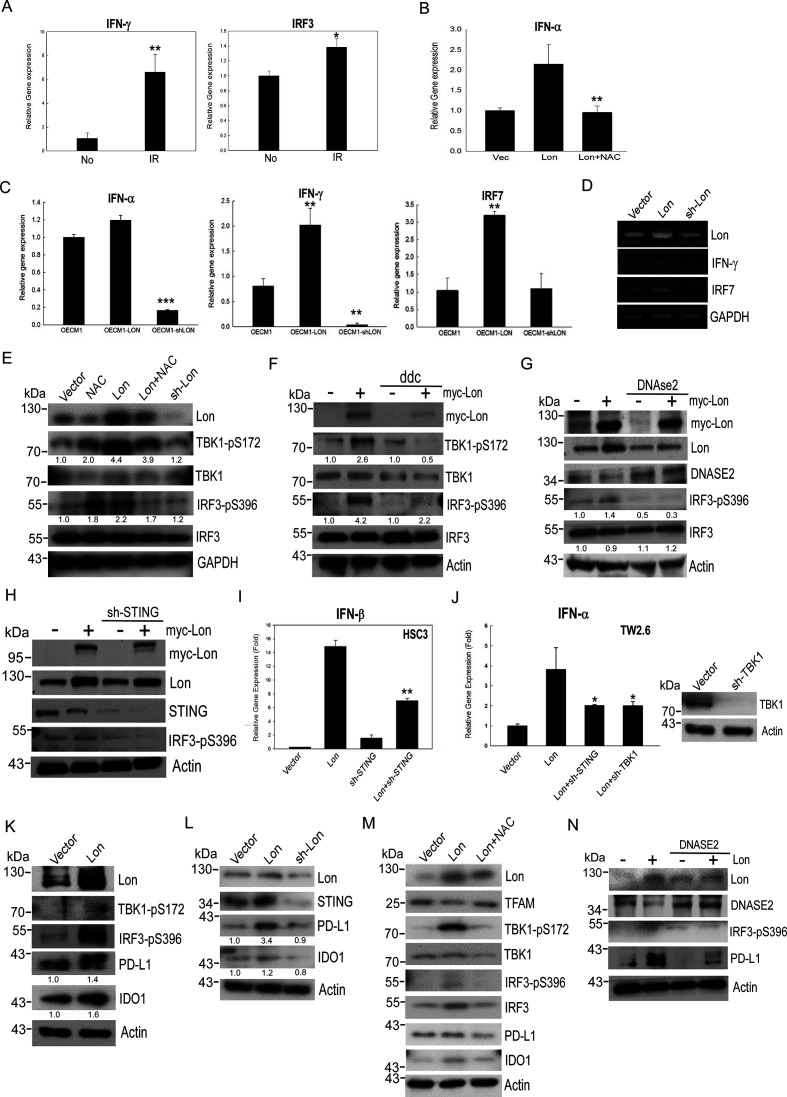
Mitochondrial Lon–mtDNA axis activates the STING-TBK1–dependent interferon (IFN) pathway in response to oxidative stress. (A) OEC-M1 cells were treated with 5 Gy IR exposure. The mRNA expressions of IFNγ and IRF3 were measured by quantitative real-time PCR. (B) OEC-M1 cells transfected with pcDNA3 plasmids encoding Lon were treated with or without NAC (5 mM, 24 hours). The mRNA expression of IFNα was measured by quantitative real-time PCR. (C, D) OEC-M1 cells were transfected with the plasmids encoding Lon or Lon-shRNA and the mRNA expressions of IFNα, IFNγ, and IRF7 were mesured by quantitative real-time PCR. (E) OEC-M1 cells were transfected with the plasmids encoding Lon or Lon-shRNA and OEC-M1-Lon cells were treated with or without NAC (5 mM) for 24 hours. The whole cell lysates were analyzed by immunoblotting using the indicated antibodies. GAPDH is used as the loading control. The number represents the band intensity normalized against GAPDH. (F) OEC-M1 cells were transfected with the plasmids encoding Lon or not and treated with or without ddC (20 µM) for 24 hours. The whole cell lysates were analyzed by immunoblotting using the indicated antibodies. Actin is used as the loading control. The number represents the band intensity normalized against actin. (G) OEC-M1 cells were transfected with the plasmids encoding Lon and/or transfected with the plasmids encoding Flag-Dnase2a. The whole cell lysates were analyzed by immunoblotting using the indicated antibodies. Actin is used as the loading control. The number represents the band intensity normalized against actin. (H) OEC-M1 cells were infected with lentivirus encoding shSTING or an empty vector. After selection with puromycin, the cells were transfected with or without the plasmids encoding myc-Lon. The whole cell lysates were analyzed by immunoblotting using the indicated antibodies. Actin is used as the loading control. (I) HSC3 cells were infected with lentivirus encoding shSTING or an empty vector. After selection with puromycin, the cells were transfected with or without the plasmids encoding myc-Lon. The mRNA expressions of IFNβ were measured by quantitative real-time PCR. (J) TW 2.6 cells were infected with lentivirus encoding shSTING or shTBK1 or an empty vector. After selection with puromycin, the cells were transfected with or without the plasmids encoding myc-Lon. The mRNA expressions of IFNα were mesured by quantitative real-time PCR. The efficiency of knocking down TBK1 was confirmed by immunoblotting. (K) OEC-M1 cells were transfected with the plasmids encoding Lon or control vector. Whole cell lysates were analyzed by immunoblotting using the indicated antibodies. Actin is used as the loading control. The number represents the band intensity normalized against actin. (L) OEC-M1 cells were transfected with the plasmids encoding Lon or Lon-shRNA. Whole cell lysates were analyzed by immunoblotting using the indicated antibodies. Actin is used as the loading control. The number represents the band intensity normalized against actin. (M) OEC-M1 cells transfected with pcDNA3 plasmids encoding Lon or control vector were treated with or without NAC (5 mM, 24 hours). Cell lysates were analyzed by immunoblotting using the indicated antibodies. Actin is used as the loading control. (N) OEC-M1 cells were infected with lentivirus encoding Flag-Dnase2a or an empty vector. After selection with puromycin, the cells were transfected with or without the plasmids encoding myc-Lon. Cell lysates were analyzed by immunoblotting using the indicated antibodies. Actin is used as the loading control.

Since mtDNA stress can trigger ISG expression via the cGAS-STING pathway in the cytosol,^11^ we hypothesized that mitochondrial Lon–ROS-induced mtDNA release into the cytosol triggers IFN signaling via a cGAS-STING-TBK1 pathway in cancer. We first confirmed whether IR irradiation induces IFN signaling and whether overexpressed Lon induces IFN expression in a ROS-dependent manner. We found that irradiation induces mRNA expression of IFN-γ and IRF3 (figure 3A) and that the expression of IFN-α was upregulated in Lon-overexpressing OSCC cells and NAC inhibited the upregulation of IFN-α expression (figure 3B). Again, the expression of IFN-α/γ and IRF7 was increased in Lon-overexpressing OEC-M1 cells; the expression was suppressed in Lon-knocked-down cells (figure 3C, D). Next, we examined whether the Lon–ROS–mtDNA axis induced the STING-TBK1 pathway in OSCC cells. The immunoblotting results showed that Lon overexpression increased the phosphorylation of TBK1 and IRF3, and the phosphorylation was decreased by the addition of ROS scavenger, NAC (figure 3E). To further verify whether Lon-induced mtDNA leakage stimulates STING-TBK1 signaling, we treated mtDNA replication inhibitor (ddC) and overexpressed DNase2a to reduce the cytoplasmic DNA in OSCC. Treatment of ddC and overexpression of DNase2a certainly decreased the levels of cytoplasmic mtDNA (figure 2F) and diminished the phosphorylation level of TBK1 and IRF3 (figure 3F and G). We then found that knocking down STING diminished Lon-induced IRF3 phosphorylation (figure 3H) and Lon-induced IFN-α/β induction (figure 3I, J) in OSCC cells. Similarly, knocking down TBK1 diminished IFN-α expression in TW2.6 cells (figure 3J). These results confirm that mitochondrial Lon-induced mtDNA release triggers IFN signaling via the STING-TBK1 pathway in OSCC. Intriguingly, we found that mitochondrial Lon also induces the expression of immunosuppressive proteins, PD-L1 and IDO1 (figure 3K, L) that are IFN-downstream genes.^34 35^ Treatment of NAC inhibited the expression of Lon-induced PD-L1 and IDO1 (figure 3M); overexpression of DNase2a reduced Lon-induced PD-L1 expression (figure 3N). Taken together, these results indicate that mitochondrial Lon–mtDNA induces the expression of IFN and ISG, such as PD-L1 and IDO1, through STING-TBK1 signaling in OSCC.

### Mitochondrial Lon induces EVs to carry mtDNA and PD-L1 from cancer cells

**Figure 4:**
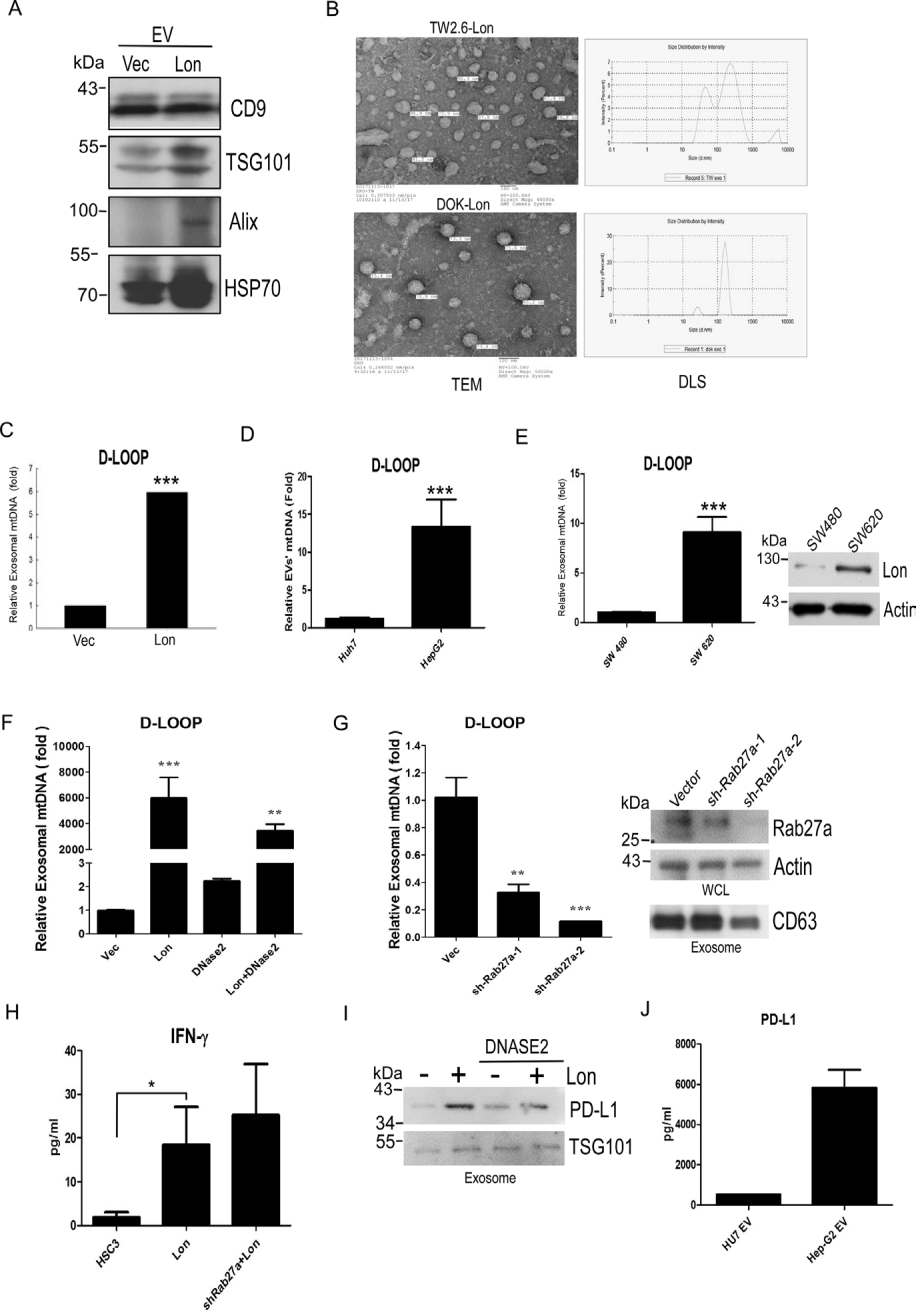
Mitochondrial Lon induces the secretion of extracellular vesicles (EVs) that carry mtDNA and PD-L1 from cancer cells. (A) Mitochondrial Lon induces EV production. DOK cells were transfected with the plasmids encoding Lon or control vector. Purified EVs were analyzed by immunoblotting using the indicated antibodies. (B) The identification of OSCC EVs was performed by transmission electron microscopy. Scale bar, 100 nm. The size distribution of oral squamous cell carcinoma EVs was performed via dynamic light scattering (right panel). (C) TW2.6 cells were transfected with the plasmids encoding Lon or control vector. EVs from the cells were purified and the relative amount of exosomal mtDNA (D-LOOP) was determined by qPCR analysis. (D) HepG2 and Huh7 cells were used to prepare EVs and the relative amount of exosomal mtDNA (D-LOOP) was determined by qPCR analysis. (E) SW480 and SW620 cells were used to prepare EVs and the relative amount of exosomal mtDNA (D-LOOP) was determined by qPCR analysis. The expression of Lon was confirmed by immunoblotting. (F) HSC3 cells were transfected with the plasmids encoding Flag-Dnase2a or control vector. After selection with hygromycin, the cells were transfected with the plasmids encoding myc-Lon. The cells were used to prepare EVs and the relative amount of exosomal mtDNA (D-LOOP) was determined by qPCR analysis. (G) HSC3 cells were infected with lentivirus encoding shRab27a or an empty vector. After selection with puromycin, the cells were used to prepare EVs and the relative amount of exosomal mtDNA (D-LOOP) was determined by qPCR analysis. The efficiency of knocking down Rab27a was confirmed by immunoblotting. The purified EVs were confirmed by immunoblotting using the indicated antibodies. Actin is used as the loading control (right panel). (H) IFNγ secretion in the culture medium from HSC3, HSC3-Lon, and HSC3-Lon/shRab27a cells was deteced by ELISA. (I) HSC3 cells were transfected with the plasmids encoding Flag-Dnase2a or control vector. After selection with hygromycin, the cells were transfected with or without the plasmids encoding myc-Lon. The purified EVs were prepared and detected by immunoblotting using the indicated antibodies. TSG101 is used as an EV loading control. (J) PD-L1 amount in the purified EVs from HepG2 and Huh7 cells was deteced by ELISA.

Previous reports have shown that ROS promote EV secretion, and ROS-damaged DNA was excreted by exosomes.^22 36^ Therefore, we tried to explore whether mitochondrial Lon induces EV secretion that contains the mtDNA. We purified the EVs from WT and Lon-overexpressing DOK, TW2.6, SW 480, and SW 620 cancer cells by using ultracentrifugation. Western blotting analysis indicated that the EVs from TW2.6 cells were identified by EV-specific markers, CD9, TSG101, Alix, and HSP70, and Lon-overexpressing TW2.6 cells induced more EV secretion than WT cells (figure 4A). The EVs were analyzed and identified for shape and size by TEM and size distribution by dynamic light scattering nanoparticle measurement system. The EVs exhibited a separated, ball-like structure and size of approximately around 100 nm (figure 4B). To determine whether Lon induced EV secretion that contains the mtDNA, we analyze the D-loop contents from the EVs. Real-time PCR analysis indicated that the EVs from Lon-overexpressed cancer cells had a 5-fold to 8-fold increase of specific mtDNA fragment of D-loop (figure 4C, D and online supplemental figure S2). Consistently, the EVs from colon cancer cells with higher Lon expression, SW620, had higher D-loop copy numbers than the ones from the cells with lower Lon expression, SW480 (figure 4E). To further confirm that Lon induced EVs containing mtDNA, we used DNAase2a overexpression and Rab27a knock-down to destroy mtDNA accumulation and translocation because DNAase2a overexpression degraded mtDNA and Rab27a is a key protein to control many steps of the exosome secretion pathway.^37^ Real-time PCR analysis indicated that exosomal mtDNA induced by Lon was reduced after DNase2a overexpression and Rab27a shRNA treatment (figure 4F and G). Consistently, IFN-γ secretion induced by Lon-mtDNA was increased after Rab27a-shRNA treatment (figure 4H), suggesting that mtDNA accumulation in cancer cells caused by Rab27a-shRNA induces STING-dependent IFN expression and secretion. Intriguingly, the immunosuppressive protein, PD-L1 was found and increased in Lon-induced EVs from cancer cells, and treatment with DNase2a decreased PD-L1 content in the EVs (figure 4I, J). These data indicate that mitochondrial Lon induces EV secretion that carried mtDNA and PD-L1 from tumor cells to affect surrounding cells in the TME.

### Mitochondrial Lon-induced EVs carrying mtDNA and PD-L1 trigger IFN and IL-6 secretion from M2 macrophages and inhibit T-cell function

**Figure 5:**
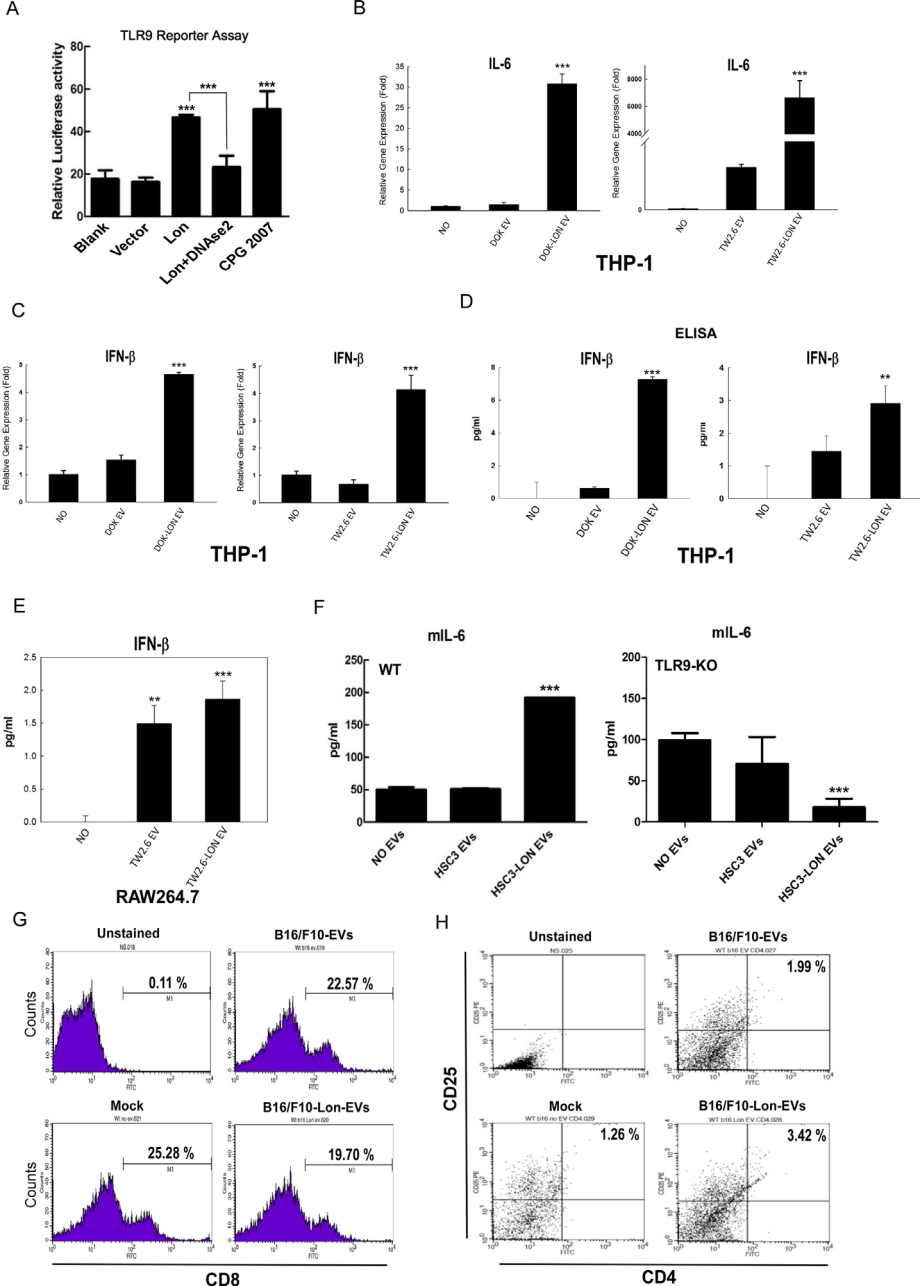
Mitochondrial Lon-induced extracellular vehicles (EVs) containing mtDNA and PD-L1 affect the behavior of macrophage and cytotoxic T cells. (A) Mitochondrial Lon-induced EVs activate the TLR9 receptor. HEK293 cells were co-transfected with a TLR9 expression vector and NF-κB controlled luciferase reporter gene overnight and then treated with the purified EVs for 7 hours with CpG 2007 (2.5 µg/mL) as a positive control. Relative luciferase activities were determined. Data are shown as mean±SD of three independent experiments. ***p<0.001. (A) Lon-induced EVs from cancer promote IL-6 expression in macrophage. THP-1 monocytes first were differentiated into macrophages using PMA (Phorbol 12-myristate 13-acetate, 100 ng/mL for 6 hours). The macrophages then were treated with the purified EVs from oral squamous cell carcinoma (OSCC) cells transfected with or without Lon plasmid for 48 hours. IL-6 mRNA expression of macrophage was detected by qPCR. (B) THP-1 monocytes first were differentiated into macrophages using PMA (Phorbol 12-myristate 13-acetate, 100 ng/mL for 6 hours). The macrophages were treated with purified EVs from transfected cells with or without Lon plasmid OSCC cells for 48 hours. IFNβ mRNA expression of macrophage was detected by qPCR. (D, E) The macrophages were incubated with the purified EVs from OSCC cells transfected with or without Lon plasmid for 48 hours. IFNβ production of macrophage was measured by ELISA. (F) Bone marrow–derived macrophages from C57BL/6 wild-type or TLR9-knocked-out mice were treated with the purified EVs from HSC3 cells transfected with or without Lon plasmid. IL-6 production of macrophage from mice was measured by ELISA. (G) Purified T cells were prepared from splenocytes of C57BL/6. Then the purified T cells were treated with the purified EVs from B16/F10 melanoma cells transfected with or without Lon plasmid or not (Mock). CD8^+^ cells were measured by flow cytometry. (H) Purified T cells were prepared from splenocytes of C57BL/6. Then the purified T cells were treated with the purified EVs from B16/F10 melanoma cells transfected with or without Lon plasmid or not (Mock). CD4^+^/CD25^+^ cells were measured by flow cytometry.

Several studies demonstrated that plasma-derived exosomes and extracellular mtDNA can be taken up by neighboring macrophages, where they activate an inflammatory response via the TLR9-NF-κB or TLR9-IFN pathways.^8 38^ Thus, we tested whether EVs containing mtDNA from Lon-overexpressed cells affect macrophage function through the TLR9-dependent pathway. First, by using the TLR9 reporter assay system, we found that EVs from overexpressed Lon cells induce higher TLR9 activity, and DNase2a treatment can reduce EV-induced TLR9 signaling, where CPG2007 acts as a positive control (figure 5A). Next, real-time PCR analysis showed that mRNA levels of IL-6 in macrophage cells THP1 are increased when THP1 cells were treated with EVs from Lon-overexpressed cells (figure 5B), suggesting that they activate an inflammatory response via TLR9-NF-κB signaling. Similarly, expression and production of IFN-β in THP1 and RAW264.7 cells are increased when they were treated with EVs from Lon-overexpressed cells (figure 5C–E). We further confirmed that the EVs containing mtDNA affect macrophage function through the TLR9-dependent pathway in vivo by using TLR-9 knocked-out (KO) mice to measure the IL-6 levels of BMDM (figure 5F). We have found that Lon expression enhances the M2 type of macrophages and suggest that secretion of immunosuppressive cytokines from macrophage may suppress T-cell function.^5^ To confirm that mitochondrial Lon-induced EV secretion that carried mtDNA and PD-L1 is involved in T-cell function suppression, we isolated T cells from splenocytes of C57BL/6 mice and treated with EVs from B16/F10 overexpressing Lon cells. The results indicated that CD8^+^ T cells are decreased when they were treated with EVs from the Lon-overexpressed cells (figure 5G). In addition, CD3^+^/CD8^+^ T cells from mice bearing tumor induced by Lon-overexpressing B16/F10 cells were decreased compared with those induced by B16/F10 cells only (9.80% vs 44.10 %) (online supplemental figure S3). We further found that CD25^+^/CD4^+^ regulatory T (Treg) cells are increased when they were treated with EVs from the Lon-overexpressed cells (figure 5H). These data show that mitochondrial Lon-induced EVs carrying mtDNA and PD-L1 either directly inhibit or indirectly inhibit T-cell function by triggering immunosuppressive cytokine secretion from M2 macrophage.

### Mitochondrial Lon-induced EVs carrying mtDNA and PD-L1 promote tumor progression in the mouse model and patients with OSCC

**Figure 6:**
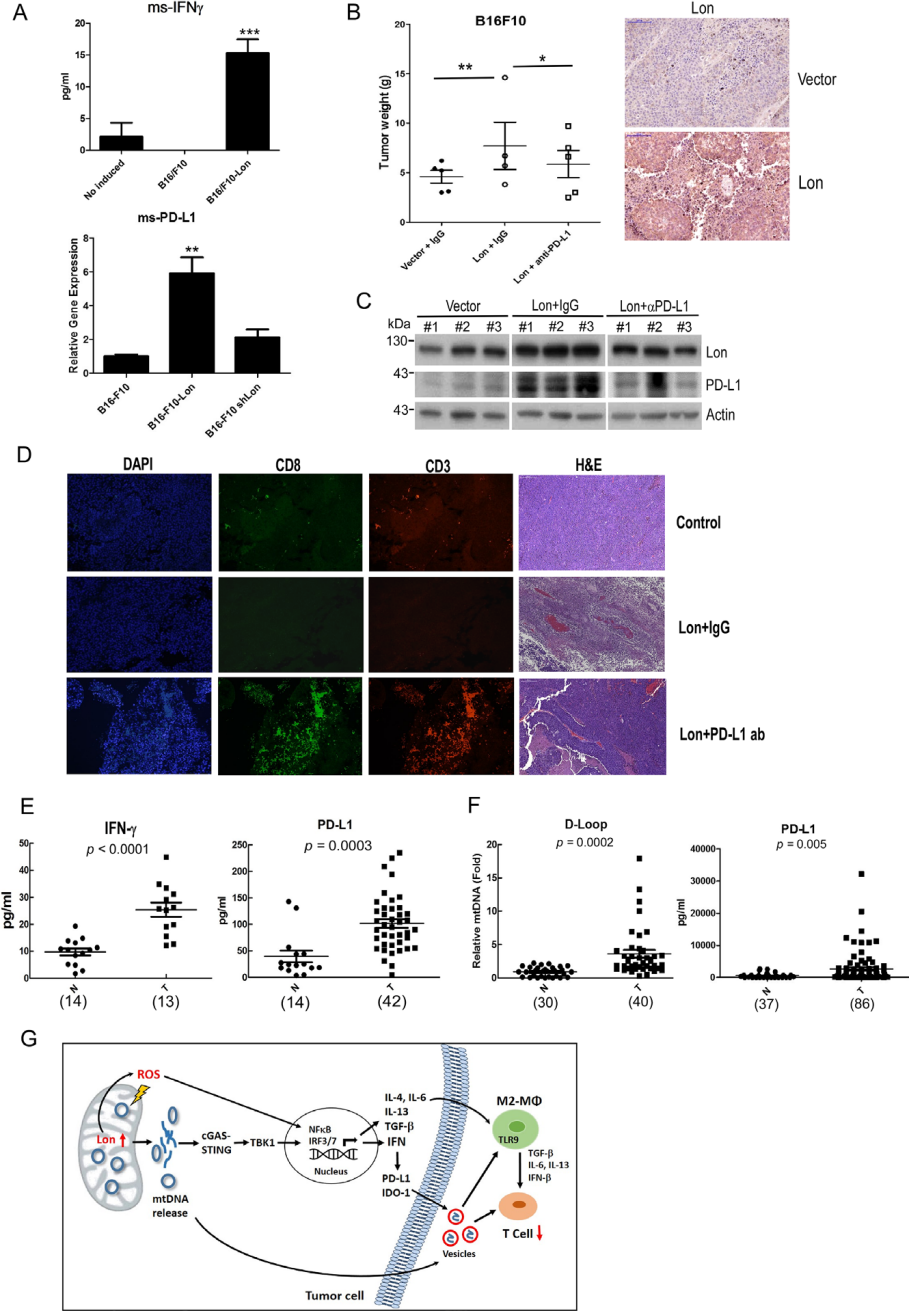
Mitochondrial Lon-induced extracellular vehicles (EVs) containing mtDNA and PD-L1 promote tumor progression in the mouse model and patients with oral squamous cell carcinoma. (A) Mitochondrial Lon induces IFN-γ and PD-L1 expression in B16/F10 melanoma. B16/F10 melanoma cells were transfected with the plasmids encoding vector, Lon, or Lon-shRNA. The transfected B16/F10 melanoma cells were injected subcutaneously into C57BL/6 mice. The msIFN-γ and msPD-L1 level in B16/F10 melanoma was determined by ELISA. (B, C) Lon overexpression promotes PD-L1–dependent tumorigenicity in vivo. B16/F10 melanoma cells were transfected with the plasmids encoding vector or Lon. The transfected B16/F10 melanoma cells were injected subcutaneously into mice. B16/F10 cells transfected with the plasmids encoding Lon were treated with IgG or anti-PD-L1 antibody (0.4 mg/kg per mouse) intraperitoneal injection (i.p.), 12 days post-inoculation and once every 3 days for three injections. The tumor volumes were measured every 3 days. Data represented are the mean of n=6 C57BL/6 mice (B). **p<0.01, *p<0.05. The tumor tissues were collected from three mice that were injected subcutaneously by the B16/F10 cells transfected with the plasmids encoding Lon or vector and treated with IgG or anti-PD-L1 antibody as indicated. Representative immunohistochemical analysis of Lon was performed by using paraffin-embedded sections of mice tissues (right panel). Scale bar, 100 µm. Whole cell lysates were analyzed by immunoblotting using the indicated antibodies. Actin is used as the loading control (C). (D) PD-L1 inhibition rescues mitochondrial Lon-reduced CD3^+^/CD8^+^ T-cell infiltration. The CD3 and CD8 immunofluorescence staining was performed in the resected tumors of C57BL/6 mice at day 17. Representative H&E staining of tumor sections from the B16/F10-Lon mouse treated with IgG or anti-PD-L1 antibody. (E) IFN-γ and PD-L1 are upregulated in plasma of patients with oral cancer. PD-L1 and IFN-γ levels were measured by ELISA in the plasma of patients with oral cancer. (F) mtDNA amount and PD-L1 are upregulated in EVs from plasma of patients with oral cancer. Relative mtDNA amount was detected by qPCR and exosomal PD-L1 level was measured by ELISA in the purified EVs from plasma of patients with oral cancer. (G) Scheme of mitochondrial Lon-induced mtDNA and PD-L1 that inhibit CD8^+^ T-cell activity in the tumor microenvironment by STING-TBK-IFN signaling and by direct secretion of EVs to attenuate anti-cancer macrophage.

To confirm that mitochondrial Lon-induced EV secretion that carried mtDNA and PD-L1 is involved in tumor progression in vivo, we injected the B16/F10 cells overexpressing Lon into B6 mice, and collected and analyzed the tumor tissues from the mice. The results indicate that Lon overexpression induces the expression of IFN-γ and PD-L1 in mouse B16/F10 cells (figure 6A). We next examined whether overexpression of Lon in cancer may have an immunosuppression effect to promote cancer progression. We confirmed the Lon-overexpressing B16/F10 cells in the xenograft mouse model and found that Lon overexpression shows an increasing volume of tumor mass and level in PD-L1, but not in the control cells and the Lon-overexpressing cells treated with PD-L1 blocker (figure 6B, C), suggesting that Lon overexpression in cancer cells promotes tumorigenicity in a PD-L1–dependent manner in vivo. Immunofluorescence staining of B16/F10–induced tumor sections revealed that the PD-L1 blocker treatment significantly rescues CD3^+^ and CD8^+^ T cells which decreases by Lon overexpression in the tumor (figure 6D). To prove the correlation of mtDNA and PD-L1 levels in the serum and EVs in plasma with tumor progression in patients with OSCC, we first screened and compared the levels in the plasma specimens from patients without cancer (N) and patients with OSCC (T) by ELISA. Compared with the normal group, plasma of patients with oral cancer showed a higher content in IFN-γ level (p<0.0001) and PD-L1 (p*=*0.0003) (figure 6E). We further confirmed that EVs of patients with oral cancer also show a higher content in mtDNA (p=0.0002) and PD-L1 (p=0.005) compared with the healthy groups (figure 6F). Subsequently, our results indicated that the level of mtDNA in plasma is significantly positively correlated with IFN-γ (p=0.0001) and PD-L1 (p=0.002) (table 1). Our results support that Lon-induced mtDNA release triggers the expression of IFN-γ/PD-L1 in OSCC, which is released into the TME via the direct secretion of EVs that inhibit T-cell activation and attnuate anti-cancer immunity.

**Table 1:** Spearman correlation coefficients between mtDNA and IFN-γ or PD-L1 in the serum specimens of patients with oral squamous cell carcinoma (n=80)

	mtDNA
IFN-γ	0.540 (0.000)
PD-L1	0.460 (0.002)

P-values are presented in parenthesis.

## Discussion

In this study, we suggest that mitochondrial Lon-induced oxidative stress persuades mtDNA damages and promotes mtDNA release into the cytosol under oxidative stress. mtDNA release induces IFN signaling through the STING-dependent pathway on oxidative stress. Furthermore, the Lon–mtDNA axis induces the expression of PD-L1 and IDO-1. Simultaneously, Lon induces the secretion of EVs that carry mtDNA and PD-L1 into the TME. Lon overexpression-induced EVs further induce the production of IFN and IL-6 from macrophages. Upregulation of the Lon–ROS–mtDNA axis causes an immunosuppressive microenvironment, which further inhibits the function of macrophages and T cells (figure 6G). Taken together, these findings suggest that mitochondrial Lon-induced mtDNA leakage and PD-L1 on EVs in OSCC is a mechanism to create an immunosuppressive microenvironment and promote tumorigenesis.

We found that the mitochondrial Lon–ROS axis promotes mtDNA damage and leakage in OSCC. Oxidative stress damages mitochondrial components such as mtDNA, protein, and lipids, which causes mtDNA mutations and mitochondria dysfunction that contribute to tumorigenesis.^39^ We provide evidence that Lon overexpression promotes the accumulation of cytosolic mtDNA with oxidative damages in oral cancer cells. However, the mechanism of how mtDNA is translocated to the cytosol is not fully clear. It has been reported that BAK/BAX macropores on the outer membrane and subsequent permeabilization of the inner membrane of mitochondria facilitate mtDNA efflux during apoptosis.^40 41^ In addition, mitochondria-derived vesicles (MDVs) may act as another way to transfer mtDNA to the cytosol of cells.^42^ Mitochondrial cargos, such as proteins, lipids, and DNA, are transported through MDVs. MDVs derive from mitochondria and function in organelle transport, regulation of mitochondrial fusion and fission, mitochondrial quality control, and immunity.^43 44^ In the future, we will examine whether mitochondrial Lon plays a role in MDV formation.

Recently, we observed that Lon-induced ROS promotes Snail-mediated EMT, angiogenesis, and metastasis by NF-κB signaling in oral cancer cells,^5 22^ indicating that mitochondrial Lon induces the ROS-dependent inflammatory response. In this work, we found that mitochondrial Lon is upregulated and triggers oxidatively damaged mtDNA release into the cytosol in response to oxidative stress. We provide the evidence that Lon overexpression promotes the inflammation by the cytosolic mtDNA-activated STING-TBK1 pathway in cancer cells and the TLR9 pathway in macrophages in the TME. Accumulating evidence suggests that mtDNA escaping from stressed mitochondria can activate inflammasomes, engage the cGAS-STING pathway to induce IFN production, and activate the TLR9 pathway to induce proinflammatory cytokine production.^8 45 46^ Stressed mitochondria and dysregulation of cytosolic DNA metabolism promote inflammation and disease development. The increased level of mtDNA damage and leakage to the cytosol induce obesity-induced inflammation^47^ and cancer metastasis.^48^ We show that the Lon-mtDNA release induces IFN signaling and further induces the expression of PD-L1 and IDO-1 on oxidative stress. Although it has been demonstrated that IFNs are critical in mounting anti-tumor response,^49^ emerging data suggested that IFN production from the cancer cells per se systemically represses the immune system.^50–52^ We found the mechanism that mitochondrial Lon-mtDNA–dependent IFN signaling suppresses CD8^+^ T-cell function is the induction of PD-L1 and IDO-1 expression.

Unexpectedly, the upregulation of mitochondrial Lon also induces the secretion of EVs, which carry mtDNA and PD-L1. However, the mechanism of the Lon-induced mtDNA release that is packed into EVs is not fully clear. We speculate that Lon-induced EVs are derived from mitochondria-derived vesicles containing mtDNA, which are secreted through mitochondria-associated membranes (MAMs)^53^ or the endosomal TLR9-mediated mitochondria–lysosome contact.^54^ Our findings indeed found that mitochondrial Lon induces the secretion of EVs containing mtDNA into the TME, which is consistent with previous reports.^36 55^ Furthermore, Lon overexpression-induced EVs further induce the production of IFN and IL-6 from macrophages in a TLR9-dependent manner, which causes an immunosuppressive microenvironment. Most interestingly, we also found that EVs from Lon-overexpressing cancer cells carry PD-L1 proteins. EVs act as a delivery messenger for cell communication in the TME. Accumulating evidence show that PD-L1 on the membrane of cancer-derived EVs contributes to immune evasion and is associated with anti-PD-1 response.^56 57^ Indeed, we demonstrated that EVs carrying PD-L1 from OSCC cells can induce the production of IFN and IL-6 from macrophages and inhibit T-cell activity. Our findings indicate that mitochondrial Lon-induced mtDNA and PD-L1 in OSCC create an immunosuppressive microenvironment via EV communication, which promotes tumorigenesis. Indeed, previous reports showed that EVs carrying PD-L1 have a correlation with immunosuppression and tumor progression in patients with glioblastoma, lung cancer, melanoma, and breast cancer.^56–58^ We further confirmed the induction of PD-L1 and IFN-γ expression and the higher levels of mtDNA contents in the plasma of patients with OSCC compared with the normal group. Therefore, we suggest that the levels of PD-L1 and mtDNA on/in EVs of plasma of patients with cancer serve as biomarker candidates for standard immunotherapy with anti-PD-L1. Immune checkpoint inhibitor therapy is the newest class of systemic cancer therapies with an improved outcome in many cancer types. However, the most severe drawback of anti-PD-L1 immunotherapy is a low response rate (20–30%), which may result from high false-negative determination of PD-L1. Our study defines a link between the mtDNA content and PD-L1 immunotherapy. We hope that the mtDNA will be used as a biomarker for anti-PD-L1 immunotherapy or in a therapy combination of anti-PD-L1 and anti-mtDNA antibody.

In summary, mitochondrial Lon-induced mtDNA release can trigger IFN production via STING-TBK1, which upregulates PD-L1 and IDO-1 expression to inhibit T-cell activation. In addition, Lon-induced EVs carrying PD-L1 and mtDNA attenuate innate and T-cell immunity in the TME. We describe a new association between the ROS–mtDNA axis and anti-PD-L1 immunotherapy in oral cancer. Thus, we suggest that the levels of mtDNA and PD-L1 in/on EVs in patients with oral cancer may function as a potential diagnostic biomarker for anti-PD-L1 immunotherapy.
