# Supplementary material for: Mitochondrial Lon-induced mtDNA leakage contributes to PD-L1–mediated immunoescape via STING-IFN signaling and extracellular vesicles
Source: J Immunother Cancer. 2020 Dec 2;8(2):e001372. doi: 10.1136/jitc-2020-001372 (PMC7713199; doi:10.1136/jitc-2020-001372)
Supplement: Supplementary data [file jitc-2020-001372supp001.pdf]

Mitochondrial Lon-induced mtDNA leakage contributes to PD-L1-mediated immunoescape via STING-IFN signaling and extracellular vesicles

An Ning Cheng[1] , Li-Chun Cheng[1], Cheng-Liang Kuo[1], Yu Kang Lo[1], Han-Yu Chou[1], Chung-Hsing Chen[1], Yi-Hao Wang[1], Tsung-Hsien Chuang[2], Shih-Jung Cheng[3,4,5], and Alan Yueh-Luen Lee[1,6]*

*: Corresponding author:

National Institute of Cancer Research, National Health Research Institutes, 35 Keyan Road, Zhunan, Miaoli 35053, Taiwan

Tel.: +886-37-206-166 ext. 31705

Fax: +886-37-586-463

E-mail: alanylee@nhri.edu.tw

ORCID ID: 0000-0003-0252-0571

Supplementary Methods

Immunofluorescence staining

HSC3 cells were plated on glass coverslips placed in a 6-well culture dish, they were washed with PBS (PBST) and then fixed with warmed 4% PFA in PBS for 15 min at room temperature. Fixed cells were washed with PBS and permeabilized with 0.3% Triton X-100 in PBS for 15 min, then blocked with 3% BSA in PBST for 1h at room temperature. Cells on coverslips were incubated with the indicated antibodies: anti-8-OHdG-FITC (1:100) and anti-TFAM (1:100) in 1% BSA in PBST overnight at 4°C. The fixed cells were washed three times by 0.3% Triton X-100 in PBST and incubated with Alexa-594 conjugated anti-rabbit secondary antibody (1:400) for 1h in dark. Finally, coverslips were mounted by ProLong Gold Antifade Reagent with DAPI (Invitrogen). Fluorescent images were acquired by the Olympus BX51 microscope.

Irradiation treatment

OEC-M1 cells were irradiated at different doses (0, 2, and 5 Gy). X-irradiation was performed with a 160 kV RS 2000 X-ray biological Irra-diator (Rad Source Technologies, USA) at a dose rate of 16.55 mGy/s at 25 mA.

Dynamic light scattering

Dynamic light scattering (DLS; Malvern ALV/CGS-3 goniometer system, ALV-GmbH, Langen/Hesse, Germany) was used to determine the size distribution profile of EVs. The

size data of EVs mentions the scattering intensity distribution (z-average) with a standard error of the mean (SE). All experiments have repeated three times.

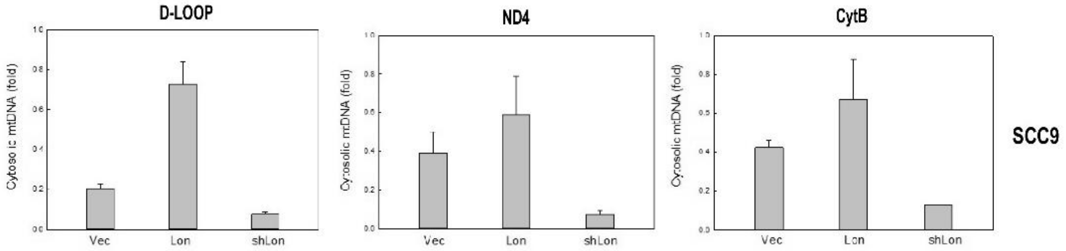

Figure S1

Figure S1. Lon overexpression promotes the accumulation of cytosolic mtDNA. Mitochondrial Lon induces mtDNA release into the cytosol in SCC9 cells. The ratio of cytoplasmic to total mtDNA was determined by qPCR analysis of SCC9 cells transfected with the plasmids encoding Lon or Lon-shRNA for 24 h (n = 3). CytB: cytochrome b; ND4: NADH dehydrogenase subunit 4.

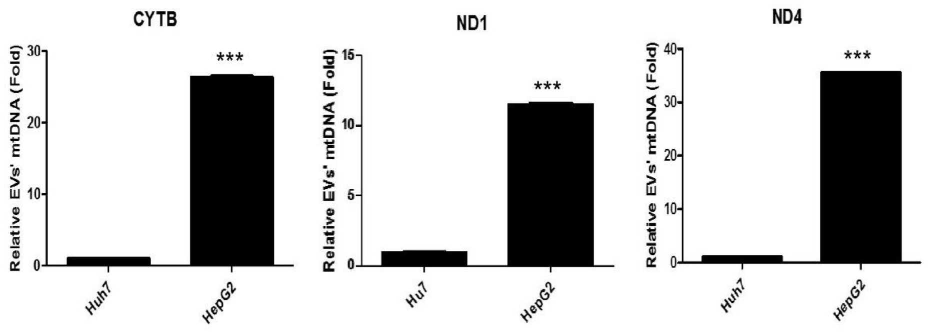

Figure S2

Figure S2. mitochondrial Lon induces the secretion of extracellular vesicles (EVs) that carry mtDNA from HCC cells.

HepG2 and Huh7 cells were used to prepare EVs and the relative amount of exosomal mtDNA (CytB: cytochrome b; ND1: NADH dehydrogenase subunit 1; ND4: NADH dehydrogenase subunit 4) was determined by qPCR analysis.

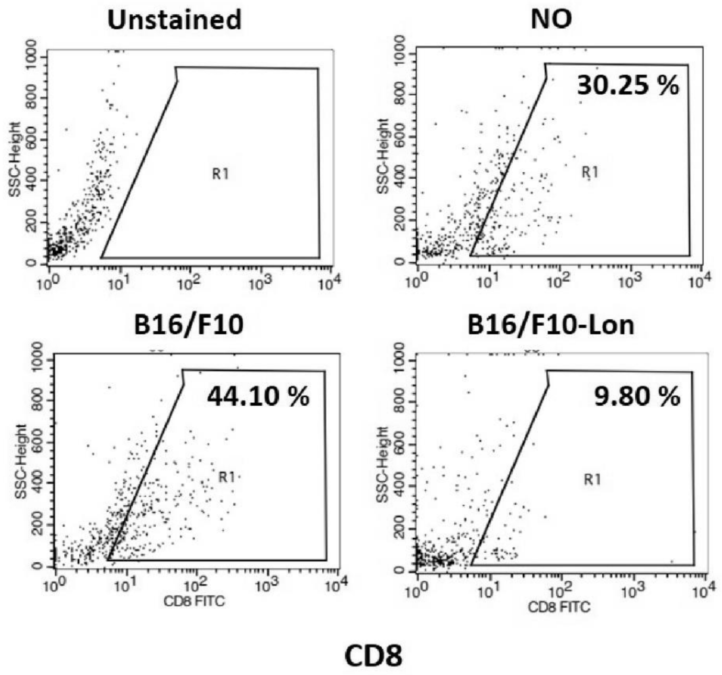

Figure S3

Figure S3. **mitochondrial Lon-induced extracellular vehicles (EVs) containing PD-L1 inhibit CD8⁺ cytotoxic T cells.** Purified T cells were prepared from splenocytes of C57BL/6. Then the purified T cells were treated with the purified EVs from B16/F10 melanoma cells transfected with or without Lon plasmid. $CD8^+$ cells were measured by flow cytometry.
